# Flexible Fe_3_O_4_@Carbon Nanofibers Hierarchically Assembled with MnO_2_ Particles for High-Performance Supercapacitor Electrodes

**DOI:** 10.1038/s41598-017-15535-x

**Published:** 2017-11-09

**Authors:** Nousheen Iqbal, Xianfeng Wang, Aijaz Ahmed Babar, Ghazala Zainab, Jianyong Yu, Bin Ding

**Affiliations:** 1 State Key Laboratory for Modification of Chemical Fibers and Polymer Materials, College of Materials Science and Engineering, Donghua University, Shanghai, 201620 China; 2 Key Laboratory of Textile Science & Technology, Ministry of Education, College of Textiles, Donghua University, Shanghai, 201620 China; 3 Innovation Center for Textile Science and Technology, Donghua University, Shanghai, 200051 China

## Abstract

Increasing use of wearable electronic devices have resulted in enhanced demand for highly flexible supercapacitor electrodes with superior electrochemical performance. In this study, flexible composite membranes with electrosprayed MnO_2_ particles uniformly anchored on Fe_3_O_4_ doped electrospun carbon nanofibers (Fe_3_O_4_@CNF_Mn_) have been prepared as flexible electrodes for high-performance supercapacitors. The interconnected porous beaded structure ensures free movement of electrolyte within the composite membranes, therefore, the developed supercapacitor electrodes not only offer high specific capacitance of ~306 F/g, but also exhibit good capacitance retention of ~85% after 2000 cycles, which certify that the synthesized electrodes offer high and stable electrochemical performance. Additionally, the supercapacitors fabricated from our developed electrodes well maintain their performance under flexural stress and exhibit a very minute change in specific capacitance even up to 180° bending angle. The developed electrode fabrication strategy integrating electrospinning and electrospray techniques paves new insights into the development of potential functional nanofibrous materials for light weight and flexible wearable supercapacitors.

## Introduction

Owing to the rapidly developing market for high performance portable electronic devices, design and fabrication of flexible energy management devices has been very critical^1–4^. In this regard, scientists are paying special attention to the supercapacitors which owe the enough potential to offer high energy density, superior power density, and excellent capacitance retention compared to dielectric capacitors and rechargeable batteries^5,6^. Various carbonaceous materials, such as graphene and carbon nanotubes etc., have been employed as active electrode materials for developing flexible supercapacitors owing to their high specific surface area and outstanding electrochemical and mechanical properties^7^. However, their high cost and complex fabrication process urge scientists to dig out alternative economical materials with superior mechanical characteristics and excellent flexible structure^8^. On the other hand, carbon nanofibers (CNF) fabricated *via* electrospinning (an easy, economic, and scalable production process^9,10^) offer utmost surface area and tailorable intra- and inter-fiber pore structure, therefore, are believed to be potentially ideal materials for developing high performance flexible supercapacitor electrodes^11^. However, pristine CNF tend to be relatively fragile in nature, therefore, certain additives for proper transmittance and scattering of external forces are needed to enhance their firmness against bending forces^12–14^.

In this regard, transition metal oxides (TMOs, compounds of oxygen bound to the transition metals), could be useful additives when employed with electrospun CNF to form nanoclusters. The uniform distribution of these nanoclusters within fiber matrix can transmit and scatter the external stresses, reduce the magnitude of the applied stress per unit area, thus, act as a barrier to crack generation and propagation^15,16^. Besides that, pore deformation and slips of graphitic carbon layers also disperse and absorb external forces, hence, support the structural flexibility of CNF^17^. Additionally, they could also be used to smartly tailor the electrochemical characteristics for developing conductive flexible electrospun CNF for portable supercapacitor applications^18,19^.

Recently, numerous TMOs (NiO^20^, CoOx^21^, MnO_2_^22^, V_2_O_5_^23^ *etc*.) offering relatively low cost, abundant availability and considerable electrochemical behaviors have been reported^24–26^. Among these TMOs, Fe_3_O_4_ is gaining special attention owing to its low cost, environment friendly nature, and readily availability^27^. However, excessive use of Fe_3_O_4_ may lead to the high volumetric changes in supercapacitors when employed as pseudo-capacitive material^28^. Additionally, its low ionic conductivity also hinders its extensive use in supercapacitors as a conducting additive^29^. Furthermore, Fe_3_O_4_ has certain limitations to be used as an electrochemical additive, however, optimized concentration of Fe_3_O_4_ may yet be very useful to tailor mechanical characteristics and flexibility of CNF without compromising their electrochemical characteristics^30,31^. Whereas, MnO_2_ besides being economical and environment friendly material, also offers excellent specific capacitance. Moreover, abundant availability and easier applicability *via* multiple fabrication processes (electrospinning, thermal decomposition, and chemical vapor deposition) make it a potential additive material for supercapacitor applications^32,33^. Therefore, a combination of Fe_3_O_4_ and MnO_2_^34^ can develop a prospective material for fabricating flexible CNF with high electrochemical performance for supercapacitor applications^35^.

In the current work, we report a highly flexible Fe_3_O_4_ doped CNF/MnO_2_ (Fe_3_O_4_@CNF_Mn_) supercapacitor electrode with superior electrochemical performance. Fabrication process initiated with electrospinning of a homogenous solution comprising of polyacrylonitrile (PAN) and Fe(acac)_3_ dissolved in dimethyl formaldehyde (DMF), followed by preoxidation and carbonization for their successful conversion into Fe_3_O_4_@CNF. The presence of Fe_3_O_4_ in CNF turned them into a highly flexible membrane. Later on, MnO_2_ deposition *via* electrospray not only enhanced the electrolyte uptake of the resultant Fe_3_O_4_@CNF_Mn_ membrane, but also boosted the overall electrochemical performance.

## Results

### Morphology and structure analysis

**Figure 1:**
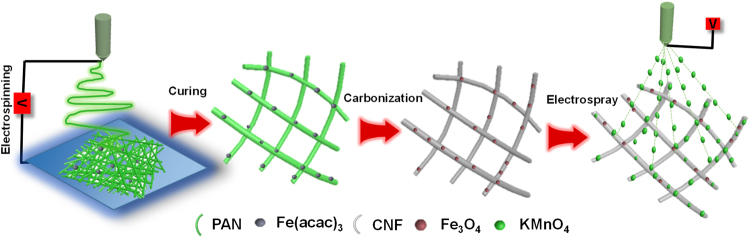
Schematic illustration of the fabrication pathway of flexible Fe_3_O_4_@CNF_Mn_.

Figure 1 demonstrates the schematic illustration of the fabrication pathway of Fe_3_O_4_@CNF_Mn_ composite membrane. At first, an identical solution comprising of PAN and iron acetylacetonate (Fe(acac)_3_) dissolved in DMF was electrospun. Resultant fibers tagged as Fe@PAN (Figure S1) were vacuum dried and stabilized by curing at 280 °C. Later on, stabilized Fe@PAN nanofibers were successfully carbonized (800 °C) under inert environment and corresponding samples were tagged as Fe_3_O_4_@CNF. Earlier, in our previous work, we figured out that pure CNF are relatively fragile in nature and have relatively low ionic conductivity and affinity towards electrolyte^11^, thus, need some metallic crystallites which may be helpful to scatter the applied forces to prevent crack development, and conductive additives to tailor their ionic conductivity and electrolyte uptake capability to obtain the desired electrochemical performance^36^. Therefore, first objective of the current work to acquire desired flexibility in CNF was achieved by inducing the regulated concentration of Fe_3_O_4_ particles in the fiber matrix which help to scatter bending stress uniformly along fiber axis, acted as a barrier to applied stress, hence, inhibited the crack propagation in the resultant fibers. Consequently, the resultant carbonized samples showed extremely high flexibility (Movie 1) when compared to pure CNF (Movie 2). Furthermore, in order to achieve second objective of high electrochemical performance, the surface of the prepared Fe_3_O_4_@CNF composite membrane was first modified with 0.1 M H_2_SO_4_ followed by electrospray of KMnO_4_^37^ (*i.e*. potential energy storage and low cost material with abundant availability and environment friendly nature), then washed with water to remove extra KMnO_4_ from the surface of the membrane and dried at 50 °C for 2 h. The electrosprayed KMnO_4_ was then converted into MnO_2_ particles *via* redox reaction^38^, which induced good ionic conductivity^39^.

**Figure 2:**
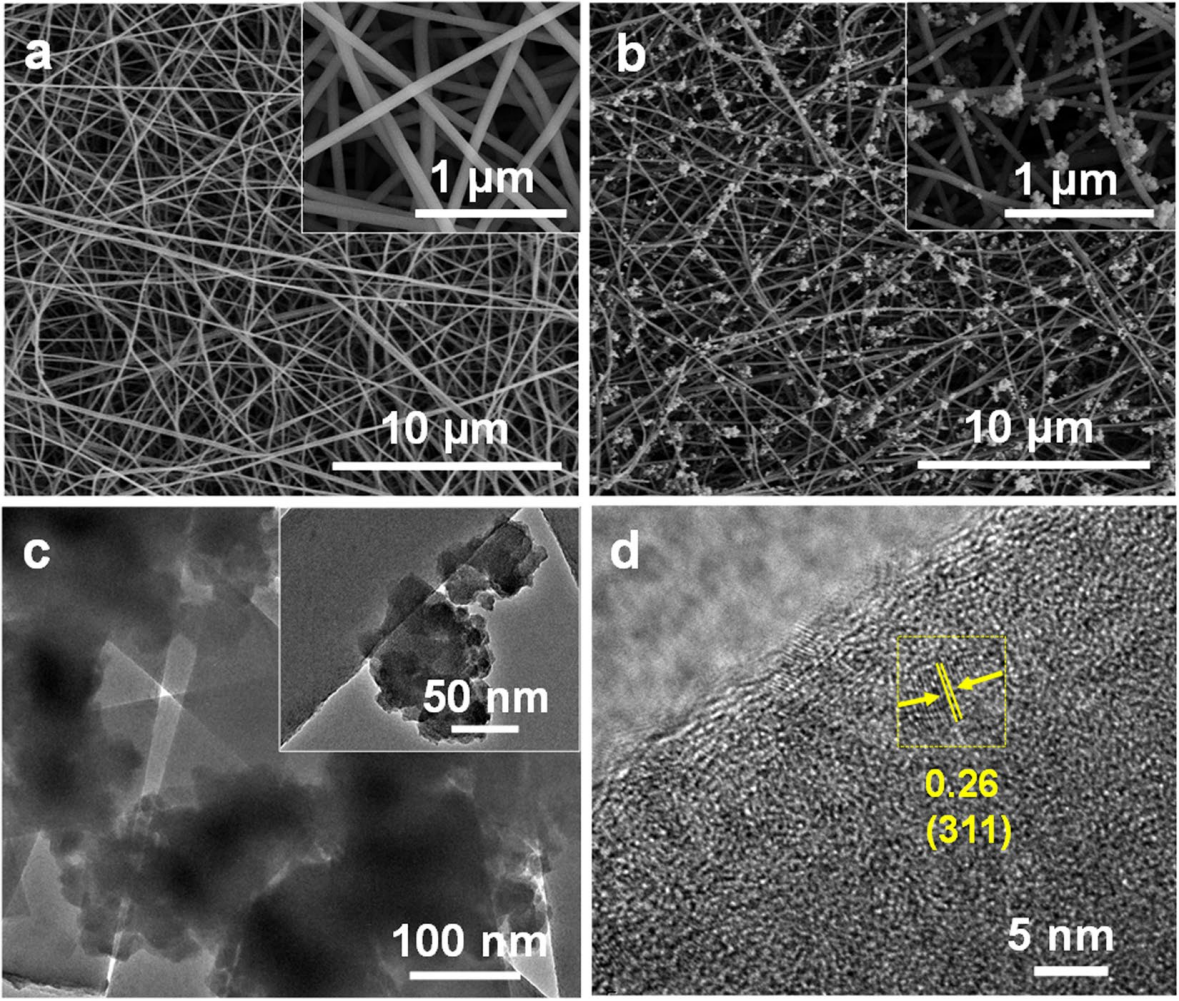
FE-SEM images of (**a**) Fe_3_O_4_@CNF and (**b**) Fe_3_O_4_@CNF_Mn_. (**c**) TEM image and (**d**) HR-TEM image of Fe_3_O_4_@CNF_Mn_.

Figure 2a,b presents the surface morphology of Fe_3_O_4_@CNF and Fe_3_O_4_@CNF_Mn_. It could be observed that both membranes offered randomly organized three dimensional fibrous structure and inherited cylindrical shaped fiber structure similar to the precursor membranes. Owing to very low concentration of Fe_3_O_4_, Fe_3_O_4_ particles were uniformly deposited inside as well as on the exterior wall of the resultant fibers (Figure S2), and did not significantly affect the fiber diameter of the subsequent Fe_3_O_4_@CNF membranes. The average fiber diameter of Fe_3_O_4_@CNF membrane ranged from 390 ± 20 nm, and the resultant membranes offered relatively low ionic conductivity, therefore, in order to achieve the desired ionic conductivity, subsequent membranes were electrosprayed with a relatively high ionic conducting material (*i.e*. MnO_2_). Random deposition of KMnO_4_ particles after redox reaction formed bead like MnO_2_ crystals on the surface of Fe_3_O_4_@CNF (Fig. 2b), enhanced the surface roughness and created open inter-particle pores on the surface of Fe_3_O_4_@CNF. Additionally, hydrophilic nature of MnO_2 _^40^ may lead to higher electrolyte uptake, thus, would improve ionic conductivity and specific capacitance of resultant Fe_3_O_4_@CNF_Mn_ composite membranes. In order to investigate the electrolyte uptake behavior, Fe_3_O_4_@CNF and Fe_3_O_4_@CNF_Mn_ were subjected to WCA examination for further analysis. Electrolyte uptake capability of the two membranes was analyzed by examining the static WCA. Fe_3_O_4_@CNF did not allow water droplets to spread much on surface and displayed a highly hydrophobic nature with the WCA of 130°, and the WCA magnificently dropped to 40° after electrospray with MnO_2_ for Fe_3_O_4_@CNF_Mn_ (Figure S3), indicating higher potential for electrolyte uptake compared to Fe_3_O_4_@CNF.

Moreover, the presence of Fe_3_O_4_ and MnO_2_ is also confirmed by TEM analysis (Fig. 2c,d). TEM image shows that nanoclusters were distributed throughout the surface of the fiber, i.e. edges as well as the body of the fiber (Fig. 2c). However, owing to deposition of a thick layer of MnO_2_ on the synthesized Fe_3_O_4_@CNF_Mn_ composite membranes, Fe_3_O_4_ sights have been overlapped with MnO_2_ crystallites, thus, are not very obvious from TEM image. Therefore, HR-TEM examination was employed to validate the presence of Fe_3_O_4_ particles in the resultant membrane (Fig. 2d), which confirmed the presence of Fe_3_O_4_ sights in the final product by showing an inter-plane distance of 2.6 Å in between the well-ordered fringes^41^. Furthermore, presence of Fe_3_O_4_ particles was also confirmed by cross-sectional SEM image (Figure S2), and close observation reveals that Fe_3_O_4_ is well dispersed in the fiber matrix with low agglomeration, hence, played lead role in developing flexible CNF.

**Figure 3:**
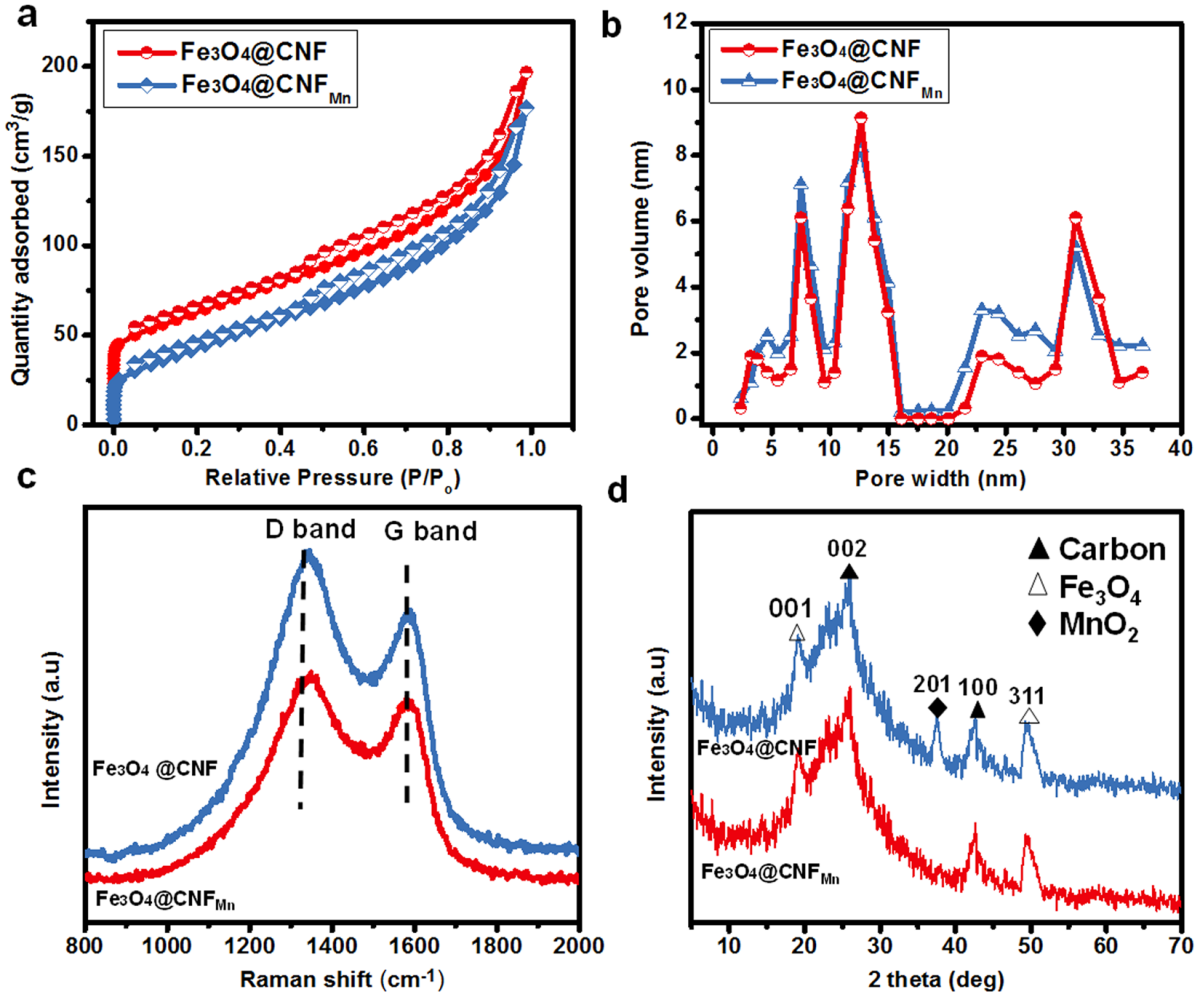
(**a**) Nitrogen adsorption/desorption isotherms, (**b**) 2D-NLDFT pore size distribution curves, (**c**) Raman spectra, and (**d**) XRD pattern of Fe_3_O_4_@CNF, and Fe_3_O_4_@CNF_Mn_ composite membrane, respectively.

Nitrogen adsorption-desorption isotherms were employed to investigate the porosity and surface area of the resultant Fe_3_O_4_@CNF and Fe_3_O_4_@CNF_Mn_ (Fig. 3a). Presence of micro- as well as mesopores for two membranes can be seen from the weak hysteresis of the typical isotherms at high relative pressure. Besides that, pore size distribution obtained by using nonlocal density functional theory (2-NLDFT) as shown in Fig. 3b, also validates the existence of micro- (Figure S4) and mesopores. Moreover, moderate BET specific surface area (~162 and ~148 m^2^/g) and considerable pore volume (~0.268 and ~0.254 cm^3^/g) for two membranes *i.e*. Fe_3_O_4_@CNF and Fe_3_O_4_@CNF_Mn_, was observed. MnO_2_ deposition probably blocked some of the surface pores resulting in a minute decrease in surface area and pore volume.

Raman spectroscopy was used for analyzing the nanostructures and degree of graphitization of the subsequent composite membranes in Fig. 3c. Two characteristic peaks at 1344 and 1580 cm^−1^ corresponding to D and G bands, respectively, show vibrations with sp^3^ bonds in the crystal lattice defects and stretching of carbon atoms bonded with sp^2^ bonds. Sp^3^ bond vibrations lead to disordered carbonaceous matrix, whereas stretching of carbon atoms bonded with sp^2^ bonds could be credited for ordered graphitized structure. Furthermore, the standard XRD patterns of Fe_3_O_4_, MnO_2_ and carbon well matched with reported diffraction peaks^19,21^, which indicate the successful development of Fe_3_O_4_ and MnO_2_ in the resultant composite CNF membrane (Fig. 3d). Moreover, the corresponding peaks at 002 and 100 represent carbon, and peaks at 001 and 311 show the presence of Fe_3_O_4_, whereas peak at 201 represent to MnO_2_. Chemical composition and structural characteristics of synthesized composite membrane are also validated by FTIR spectra (Figure S5). The characteristic peaks at 468, 518 and 1405 cm^−1^ correspond to Mn-O stretching, Fe-O stretching and C-H stretching, respectively, whereas peaks at 1163 and 2965 cm^−1^ represent the stretching mode of C-O and N-H, respectively.

**Figure 4:**
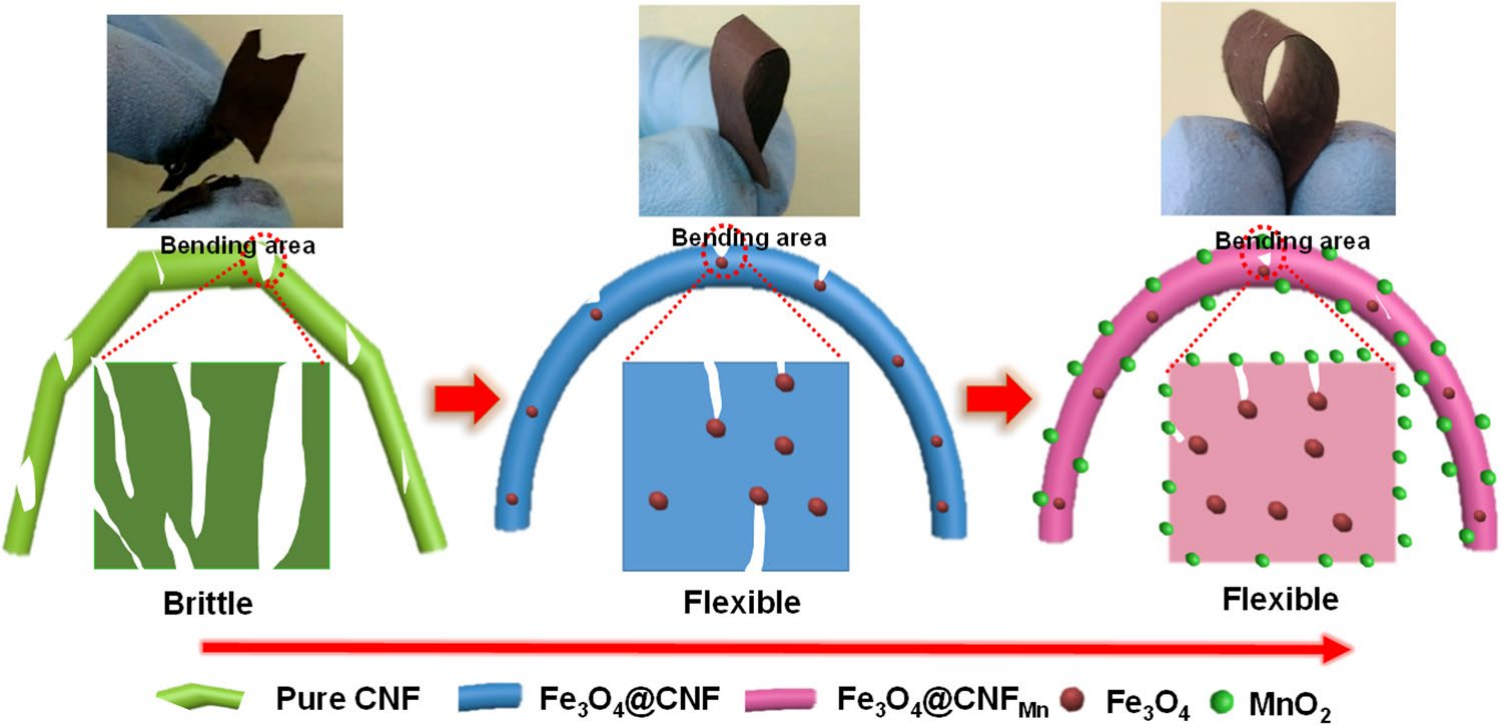
Schematic illustration showing the structure and probable mechanism of the flexibility of Fe_3_O_4_@CNF_Mn_.

Based on the systematic analysis of the nanostructures of CNF membranes, the possible mechanism behind enhanced flexibility of synthesized CNF is demonstrated in Fig. 4. Ordered distribution of the Fe_3_O_4_ and ordered graphitized carbon layers in the fiber matrix form a hierarchical composite nanostructure. Owing to the interconnected net like structure of CNF membranes, the external stress on the CNF membranes would lead to structural deformation of single fibers. Bending stresses remain condensed on the subjected area in the pure CNF, therefore, rapidly overcome their tolerance against external stress and result in formation and propagation of cracks leading to the complete fracture of membrane^42^. Whereas, incorporation of the Fe_3_O_4_ crystallites and MnO_2_ beads distribute the applied bending stresses and scatter them rapidly in fiber axis, hence, magnitude of the force per unit area is decreased^43^. Moreover, this deposition of Fe_3_O_4_ and MnO_2_ also absorb some of the applied forces and act as buffer to stop extension of tiny cracks. Therefore, composite CNF can withstand higher magnitude of bending forces and show high flexibility.

### Electrochemical performance

The electrochemical performance of the developed membranes was evaluated by using galvanostatic charge/discharge (GCD), cyclic voltammetry (CV), and electrochemical impedance spectroscopy (EIS). Since the sole purpose of using metal particles was to develop flexible carbon nanofibers, therefore, optimized quantity of metallic particles was incorporated in the current work to induce flexibility and avoid volumetric changes in the synthesized CNF. Thus, Fe_3_O_4_ presence had just negligible effect on the electrochemical performance of the synthesized membrane which is also evident from Figure S6.

**Figure 5:**
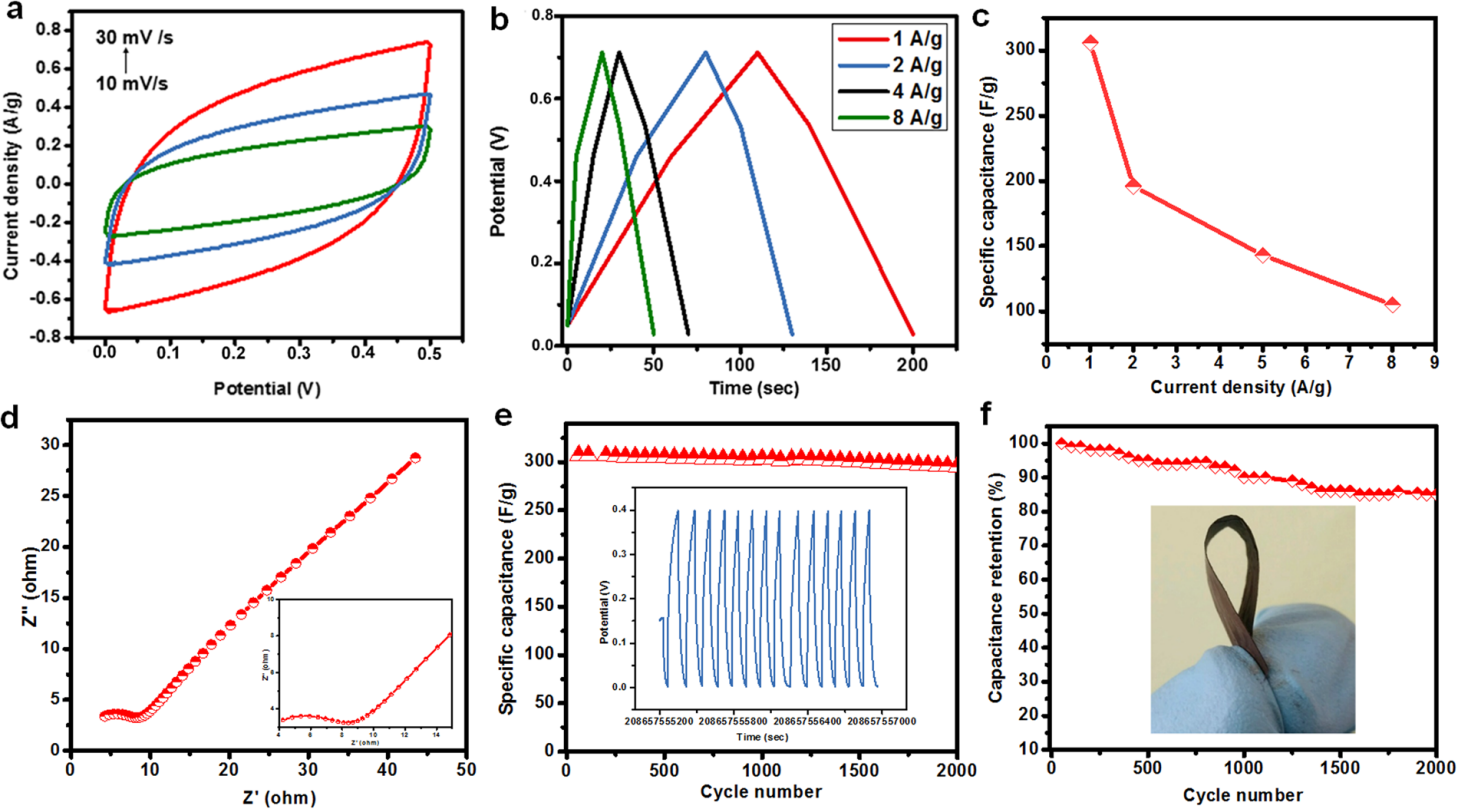
(**a**) CV, (**b**) GCD curves at 1–8 A/g, (**c**) Specific capacitance with respect to current density, (**d**) Nyquist impedance plots, (**e**) Cyclic stability analysis (the inset shows GCD curves at 0.5 A/g for 15 cycles), and (**f**) Specific capacitance retention of Fe_3_O_4_@CNF_Mn_ electrodes, inset show the digital image of flexible Fe_3_O_4_@CNF_Mn_ electrode.

Figure 5a presents the CV curves of the Fe_3_O_4_@CNF_Mn_ at various scan rates (10–30 mV/s) in the voltage range of 0–0.5 V. To further elucidate the electrochemical behavior of the Fe_3_O_4_@CNF_Mn_, GCD test was carried out at various current densities 1–8 A/g (Fig. 5b and Figure S7). GCD curves in the potential range endorsed the high charge-discharge reversibility, however, specific capacitance for two membranes decreased with increment of current densities (Fig. 5c). Specific capacitance values obtained for Fe_3_O_4_@CNF were 120, 98 F/g at 1 and 2 A/g (Figure S8), and for Fe_3_O_4_@CNF_Mn_ were 306, 196, 143, 103 F/g at rising current densities of 1, 2, 4 and 8 A/g, respectively (Fig. 5c). It is worth noting that only a minute MnO_2_ loading (39 mg/g) on Fe_3_O_4_@CNF could result in the highest specific capacitance of ~306 F/g at the current density of 1 A/g. Furthermore, specific capacitance values for corresponding GCD curves (equation 1) for Fe_3_O_4_@CNF_Mn_ are not only relatively much higher than Fe_3_O_4_@CNF but also higher than many of recently reported works^30,44^. A comprehensible comparison of current work with previous reported works is illustrated in Table S1. The specific capacitance of these flexible Fe_3_O_4_@CNF_Mn_ can be calculated by using the following equation:

1 $${\boldsymbol{C}}=\frac{{\boldsymbol{I}}{\rm{\Delta }}{\boldsymbol{t}}}{{\boldsymbol{m}}{\rm{\Delta }}{\boldsymbol{V}}}$$

where, I, Δt, m and ΔV represent current, discharge time, mass of electrodes, and voltage, respectively.

Furthermore, electrochemical impedance spectroscopy (EIS) was used to comprehend the mechanism responsible for such an excellent specific capacitance performance of Fe_3_O_4_@CNF_Mn_ composite membrane, and is represented by the Nyquist plot. As shown in Fig. 5d, Nyquist plot could be differentiated into two regions, i.e. semicircle located in high-frequency region and Nyquist curve (Warburg impedance) in the low frequency region. Semicircle represents the electrolyte resistance (resistance of electrode and solution), therefore, smaller the diameter of semicircle in the EIS spectrum, the lower would be the charge-transfer resistance. Inset of Fig. 5d shows the magnified image of semicircle region. The straight line witnessed along the imaginary axis corresponds to the polarizable capacitance^15^. Moreover, no significant IR drop in GCD curves also validates the ideal capacitive behavior of the synthesized composite membrane which could be credited to the higher ionic conductivity and relatively improved electrolyte uptake as compared CNF^45^.

**Figure 6:**
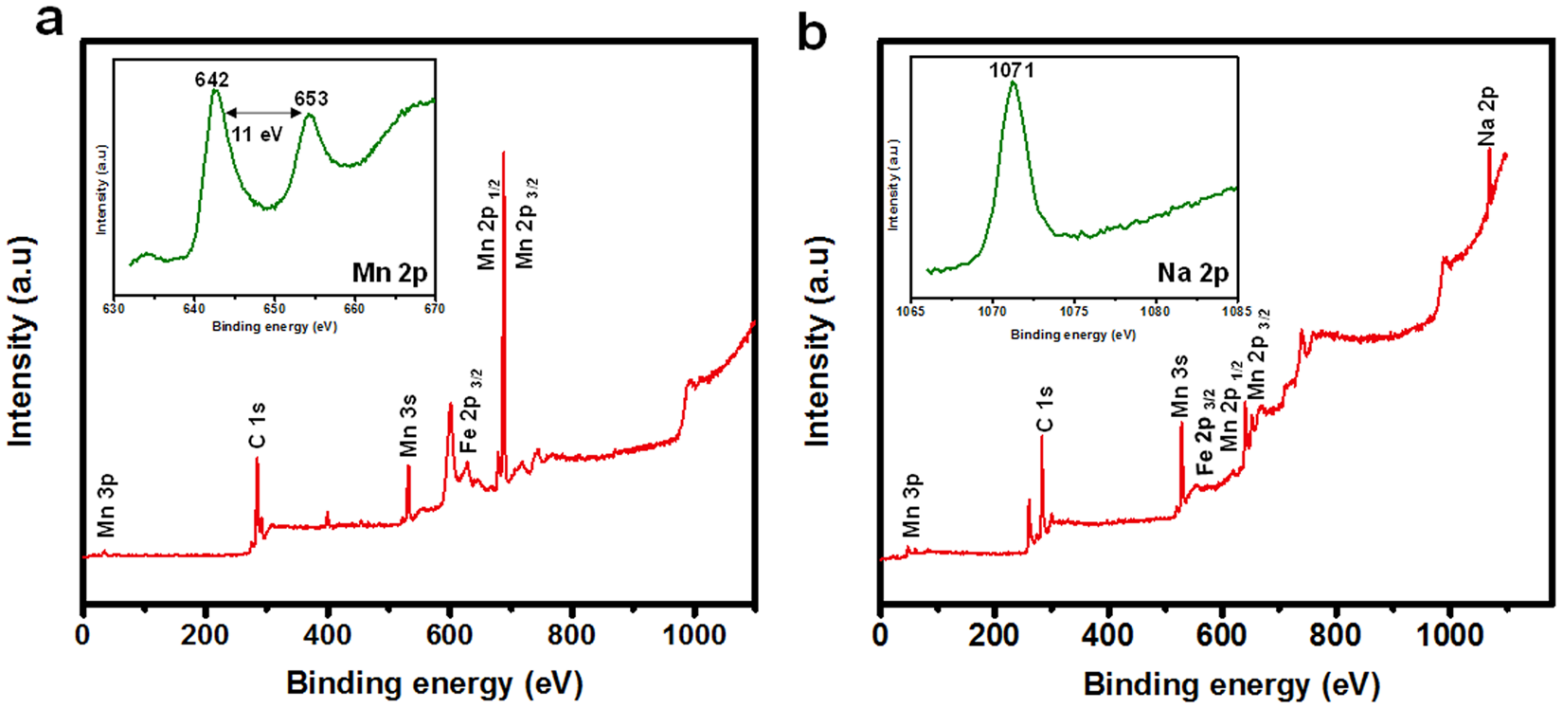
XPS spectra of Fe_3_O_4_@CNF_Mn_ (**a**) before and (**b**) after 2000 cycles. The insets show spectra of Mn 2p and Na 2p.

Figure 5e illustrates the variation in specific capacitance with respect to GCD cycle numbers, minute decrease in specific capacitance for the developed composite membranes was observed for first 300 cycles which could be attributed to the linear activation of Fe_3_O_4_ and MnO_2_, however, there was enough change observed in specific capacitance after 2000 cycles, but was not enough to alter the performance of supercapacitor (Fig. 5e, inset presents the corresponding GCD curves of 15 cycles). Additionally, prepared membranes showed reasonably high capacitance retention (*i.e*. ~85% of its initial value) after 2000 cycles (Fig. 5f, inset shows the digital image of flexible Fe_3_O_4_@CNF_Mn_ electrode), however, reason of 15% drop in specific capacitance could be credited to the MnO_2_ dissolution, oxygen evolution reaction (common drawback of MnO_2_) and structural deformation at nanoscale. In order to confirm structural deformation at nanoscale, samples were further subjected to FTIR and XPS examinations after cyclic performance. FTIR analysis of Fe_3_O_4_@CNF_Mn_ after cyclic performances showed that there was obvious decrease in the 468 cm^−1^ region which confirmed the MnO_2_ dissolution (Figure S5). Elemental analysis via XPS also validated the above findings. Existence of C, O, Fe and Mn in the Fe_3_O_4_@CNF_Mn_ samples (before and after cycles) could be endorsed from Fig. 6. High resolution XPS spectra of individual elements are illustrated in Figure S9. The binding energy separation of 11 eV between the peaks at 653 and 642 eV attributed to Mn 2p_1/2_ and Mn 2p_3/2_, respectively, and separation energy of 4.9 eV of Mn 3 s spin orbit doublet^45^ confirmed the intermediate state of Mn (Figure S9). It could be observed that Mn 2p_1/2_ and Mn 2p_3/2_ peaks declined after cyclic performance which validated the structural deformation of Mn at nanoscale, thus, may be credited for 15% loss of specific capacitance for after 2000 GCD cycles.

**Figure 7:**
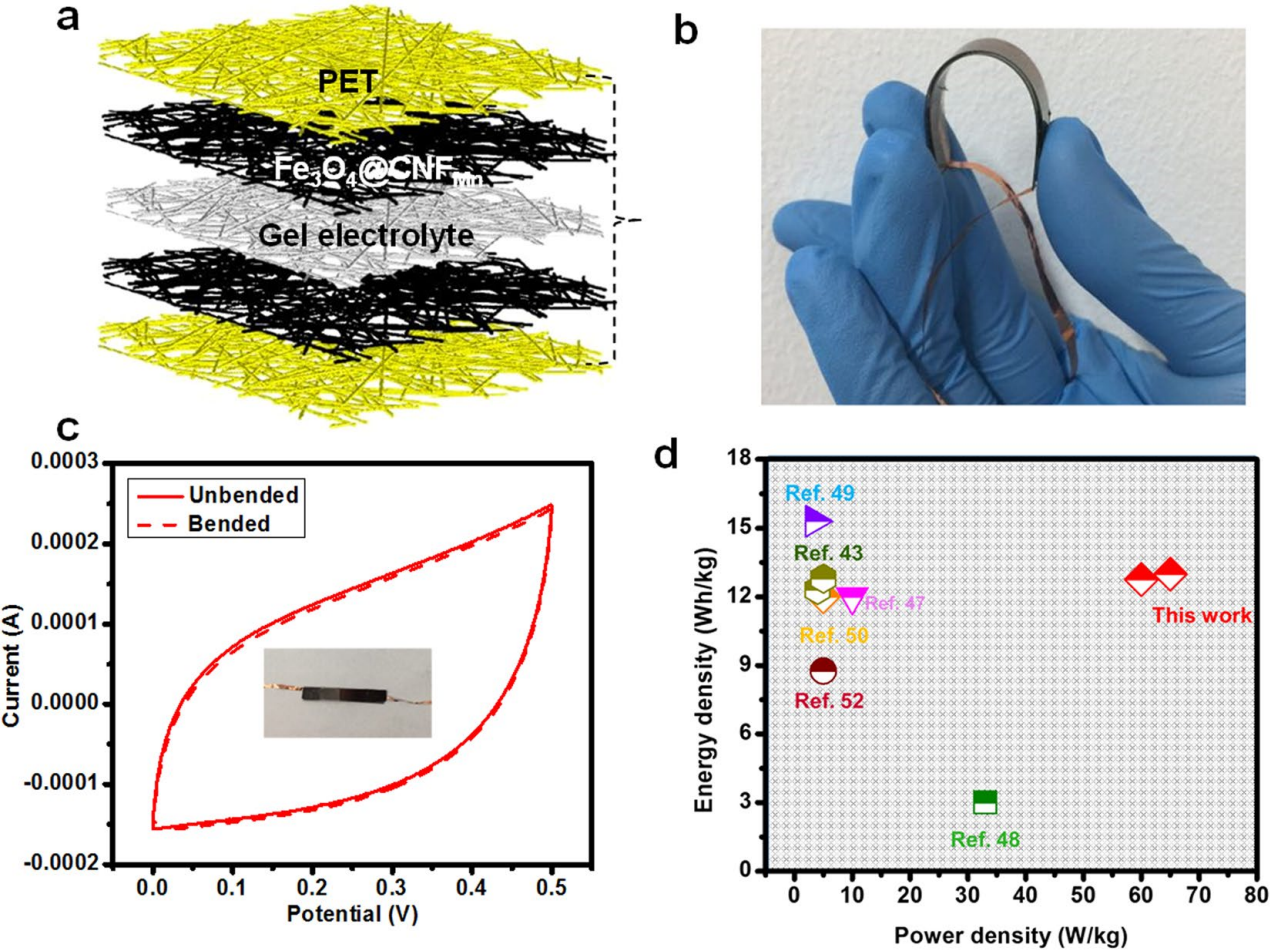
(**a**) Demonstration of fabricated supercapacitor device, (**b**) Digital image of the fabricated supercapacitor assembly, (**c**) CV curves collected at a scan rate of 20 mV/s at 180° angle of Fe_3_O_4_@CNF_Mn_, (**d**) Comparison of power and energy density of the fabricated supercapacitor device with recently reported devices.

The fabricated supercapacitor device comprised of gel electrolyte in the center covered with Fe_3_O_4_@CNF_Mn_ composite membrane on each side followed by a gold sputtered polyethylene terephthalate (PET) protective layer (Fig. 7a). As the objective was to design flexible supercapacitors, therefore, feasibility of optimized samples (Fe_3_O_4_@CNF_Mn_) for developing flexible supercapacitor devices was evaluated by investigating the specific capacitance of the fabricated symmetric supercapacitor device when subjected to bending force (Fig. 7b). No significant change in specific capacitance for optimized electrode even at bending angle of 180° was figured out (Fig. 7c), which confirmed that the synthesized composite nanofiber membranes have enough potential to fabricate flexible supercapacitors and could be used for wearable applications. Additionally, physical stability against flexural forces was also investigated using bending deformation cycles to determine the extent of flexibility of developed supercapacitors (Figure S10). It was figured out that even after 1000 bending cycles at 180°, there was no significant change in specific capacitance as well as physical structure of samples which ensured long and stable life of synthesized membrane.

Energy and power densities are two key characteristics which determine the energy storage and charging potential of supercapacitors, and are calculated by following equations.

2 $${\boldsymbol{E}}=\frac{1}{2}{\boldsymbol{C}}{{\boldsymbol{V}}}^{2}$$

3 $${\boldsymbol{P}}=\frac{{\boldsymbol{E}}}{{\rm{\Delta }}{\boldsymbol{t}}}$$

where, E is energy density, C represents specific capacitance, V is the cell voltage, P is power density, and Δt is the discharging time.

Figure 7d illustrates a Ragone plot (energy density *vs* power density) for the Fe_3_O_4_@CNF_Mn_ composite membrane supercapacitor and compares the currently fabricated supercapacitors with earlier reported works^43–52^. It could be noticed that the fabricated supercapacitors from Fe_3_O_4_@CNF_Mn_ demonstrated relatively high power density (~65 W/Kg) at high energy density (~13 Wh/kg) which is superior to many of the reported supercapacitors. This may be attributed to the stable electrolyte transport, superior surface area and relatively high number of micro- and mesopores, which provided a number of channels for easier ionic charge transport, thus improved the conductivity of the Fe_3_O_4_@CNF_Mn_ composite membrane and high power density.

## Discussion

Fe_3_O_4_@CNF_Mn_ composite nanofiber membrane has been successfully developed with bead on string structure by the combination of electrospinning and electrospray processes. The resultant membrane offered well scattered Fe_3_O_4_ particles within membrane matrix which turned the fragile nature of CNF into highly flexible structure, and MnO_2_ particles deposition throughout the membrane surface enabled fast and reversible faradic reaction and supplied shorter path for ionic diffusion. 3D micro-mesoporous structure of MnO_2_ provided larger contact area for electrode and electrolyte, supported higher electrolyte uptake, and also helped in scattering the external stress applied on the membrane to prevent membrane distortion. Quantitative performance analysis of synthesized nanofiber composite membrane showed that the membranes owed highly capacitive behavior which was apparent from rectangular shaped CV curves. Increase in current density decreased specific capacitance, whereas nonlinear behavior of charge discharge curves confirmed the contribution of pseudo capacitive mechanism. High conductivity and electrolyte uptake capacity owing to micro- and mesopores of the membrane leaded to an excellent specific capacitance of ~306 F/g. The high capacitance retention of ~85% even after 2000 cycles determined long and stable life of fabricated device. Flexibility of the synthesized membrane was further confirmed when fabricated supercapacitor was subjected to bending up to 180° and no significant change in specific capacitance was observed for up to 1000 bending cycles. Consequently, it was concluded that this technique offers a novel, valuable and scalable route for the designing flexible high-performance supercapacitors.

## Experimental Section

### Materials

Polyacrylonitrile (PAN, Mw = 90,000), polyvinyl alcohol (PVA, Mw = 86,000), Potassium hydroxide (KOH), Fe(acac)_3_, and KMnO_4_ were purchased from Aladdin Chemicals Co. Ltd., China. Dimethylformamide (DMF), and sulphuric acid (H_2_SO_4_), were provided by Shanghai Chemical Reagents Co., Ltd., China. All chemicals were used without further purification.

### Fabrication of the Fe_3_O_4_@CNF membrane

A homogenous solution comprising of Fe(acac)_3_ (1 wt.%) and PAN (8 wt.%) dissolved in DMF by magnetic stirring was electrospun following same conditions as reported in our earlier work^18^. Resultant precursor fibers were then vacuum dried and pre-oxidized at 280 °C for 2 h followed by carbonization at 800 °C for 2 h with a heating rate of 2 °C/min under nitrogen environment. Developed membrane showed ~50 µm thickness after treatment.

### Surface Modification and Electrospray of KMnO_4_

Prior to KMnO_4_ electrospray, Fe_3_O_4_@CNF composite membrane was rinsed with 0.1 M H_2_SO_4_ and washed with water then dried for 1 h at 50 °C. Typically, 5 wt.% dispersion of KMnO_4_ with PVA was electrosprayed directly onto subsequent surface modified Fe_3_O_4_@CNF using same electrospinning setup. A DC voltage of 15 kV was applied at a feeding rate of 1 mL/h and a distance of 20 cm between tip of the needles and Fe_3_O_4_@CNF (placed on stationary metallic cylinder) was maintained. Constant temperature and relative humidity were maintained at 23 ± 2 °C and 50 ± 5%, respectively. Later on, heat treatment was applied on resultant membrane to form MnO_2_ on the surface as shown in equation^45^.

4 $${\boldsymbol{Mn}}{{{\boldsymbol{O}}}_{{\bf{4}}}}^{-}+{\bf{4}}{{\boldsymbol{H}}}_{{\bf{2}}}{\boldsymbol{O}}\to {\boldsymbol{Mn}}{{{\boldsymbol{O}}}_{{\bf{2}}}}^{{\bf{2}}-}+{\bf{3}}{{\boldsymbol{O}}}_{{\bf{2}}}+{\bf{2}}{{\boldsymbol{H}}}_{{\bf{2}}}{\boldsymbol{O}}$$

5 $${\bf{4}}{\boldsymbol{Mn}}{{{\boldsymbol{O}}}_{{\bf{4}}}}^{-}+{\bf{3}}{\bf{C}}+{{\boldsymbol{H}}}_{{\bf{2}}}{\boldsymbol{O}}\to {\bf{4}}{\boldsymbol{Mn}}{{\boldsymbol{O}}}_{{\bf{2}}}+{\boldsymbol{C}}{{{\boldsymbol{O}}}_{{\bf{3}}}}^{{\bf{2}}-}+{\bf{2}}{\boldsymbol{HC}}{{{\boldsymbol{O}}}_{{\bf{3}}}}^{-}$$

### Structural Characterization

Field emission scanning electron microscope (FE-SEM, S-4800, Hitachi Ltd. Japan), high resolution transmission electron microscopy (HR-TEM, JEM-2100F, JEOL Ltd., Japan) and Raman spectroscopy system (invia-Reflex, Renishaw, Co., UK) were employed for morphological and structural analysis. X-ray diffraction (XRD) (D/Max-2550 PC Rigaku Co., Japan, Cu Kα, λ = 1.5406 Å) was involved for phase structural analysis and an automatic adsorption system (ASAP 2020, Micromeritics Co., USA) was used for examining Brunauer-Emmet-Teller (BET) surface area and porous structure by N_2_ adsorption-desorption isotherms. X-ray photoelectron spectroscopy (XPS) analysis was carried out on a PHI 5000 C ESCA system with Mg Kα source. Water contact angle (WCA) was tested by using a digital goniometer (Kino SL200B).

### Electrochemical Measurement

Electrochemical workstation (Chenhua CHI 660E, Shanghai) was employed for all electrochemical measurements. Two electrode setup was used to evaluate electrochemical performance of the all the samples. Prepared membranes were used directly employed as electrodes (each electrode weighed ~1 mg) to fabricate two electrode symmetric coin cells. Gel electrolyte (Na_2_SO_4_/PVA) was used as separator between two electrodes for all measurements. Moreover, in order to evaluate flexibility, each electrode was followed by gold sputtered polyethylene terephthalate (PET) on external side as protective layer. Cyclic voltammetry (CV) tests were carried out at various scan rates (*i.e*. 10–30 mV/s). Galvanostatic charge–discharge tests were analyzed using Chronopotentiometry (CP) at 1–8 A/g current density. Voltage drop of the synthesized device was calculated by charging at 0.5 V for 15 min followed by measuring the open circuit potential.

## Electronic supplementary material

Supporting Information

Movie 1

Movie 2
