# Supplementary material for: Flexible Fe3O4@Carbon Nanofibers Hierarchically Assembled with MnO2 Particles for High-Performance Supercapacitor Electrodes
Source: Sci Rep. 2017 Nov 9;7:15153. doi: 10.1038/s41598-017-15535-x (PMC5680204; doi:10.1038/s41598-017-15535-x)
Supplement: Supplementary file 1 — Supporting Information [file 41598_2017_15535_MOESM1_ESM.pdf]

Electronic Supplementary Information (ESI)

Flexible Fe_3O_4@Carbon Nanofibers Hierarchically Assembled with MnO_2 Particles for High-Performance Supercapacitor Electrodes

Nousheen Iqbal,[1,2] Xianfeng Wang*,[1,2,3] Aijaz Ahmed Babar,[1] Ghazala Zainab,[1] Jianyong Yu,[2] and Bin Ding*,[1,2,3]

[1]State Key Laboratory for Modification of Chemical Fibers and Polymer Materials, College of Materials Science and Engineering, Donghua University, Shanghai 201620, China.

[2]Key Laboratory of Textile Science & Technology, Ministry of Education, College of Textiles, Donghua University, Shanghai 201620, China.

[3]Innovation Center for Textile Science and Technology, Donghua University, Shanghai 200051, China.

***Corresponding author**: Prof. Xianfeng Wang, Prof. Bin Ding

E-mail address: wxf@dhu.edu.cn (X. Wang), binding@dhu.edu.cn (B. Ding)

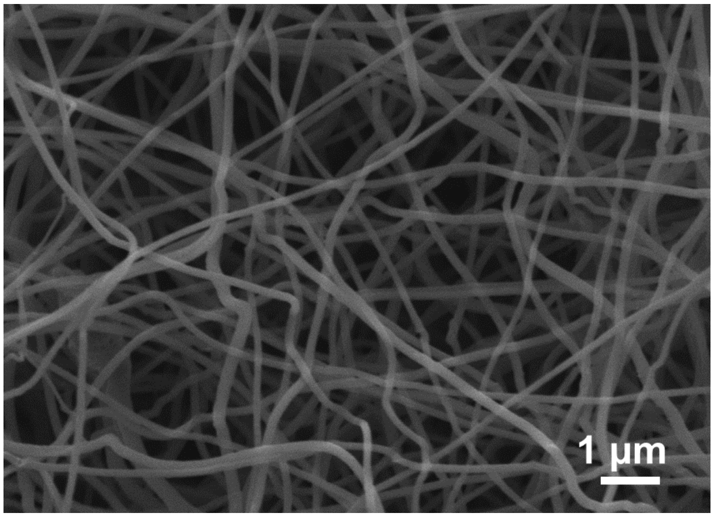

Figure S1 SEM image of Fe_3O_4@PAN.

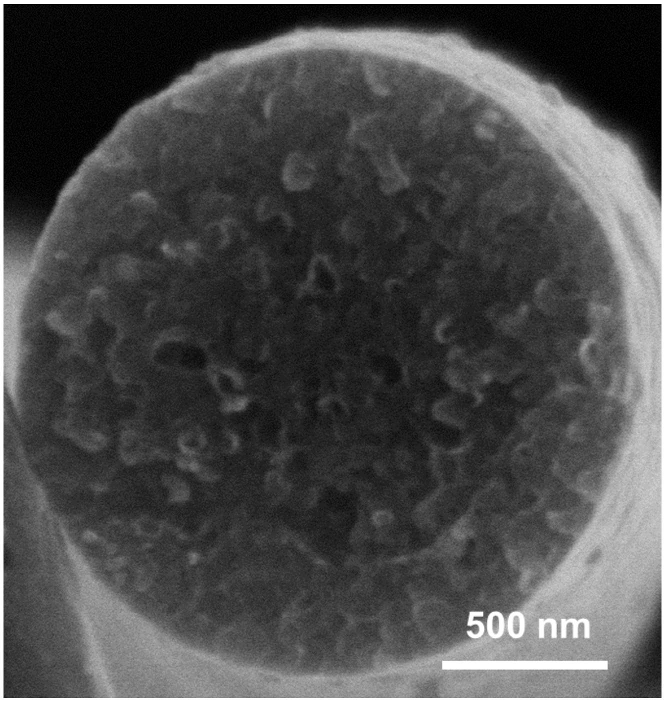

Figure S2 Cross-sectional FE-SEM image of Fe_3O_4@CNF.

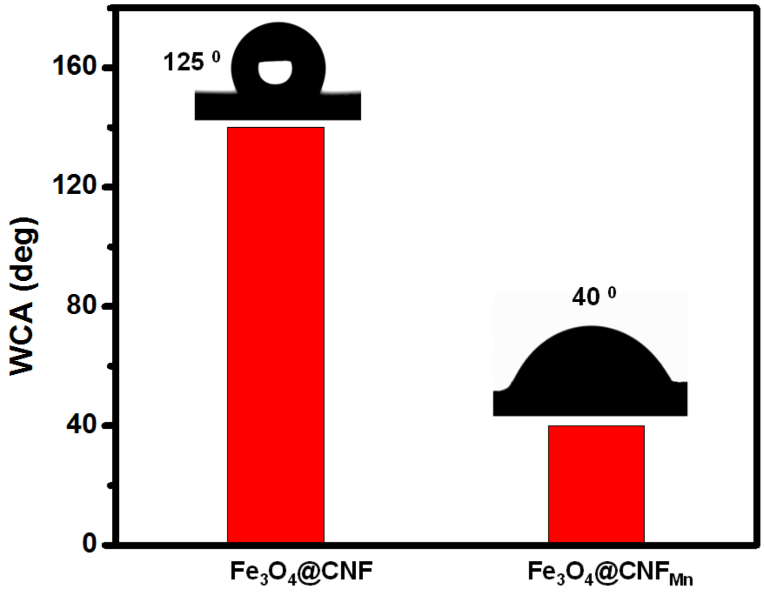

Figure S3 The behavior of water droplet moving towards the surface of (a) Fe_3O_4@CNF and (b) Fe_3O_4@CNF_{Mn}

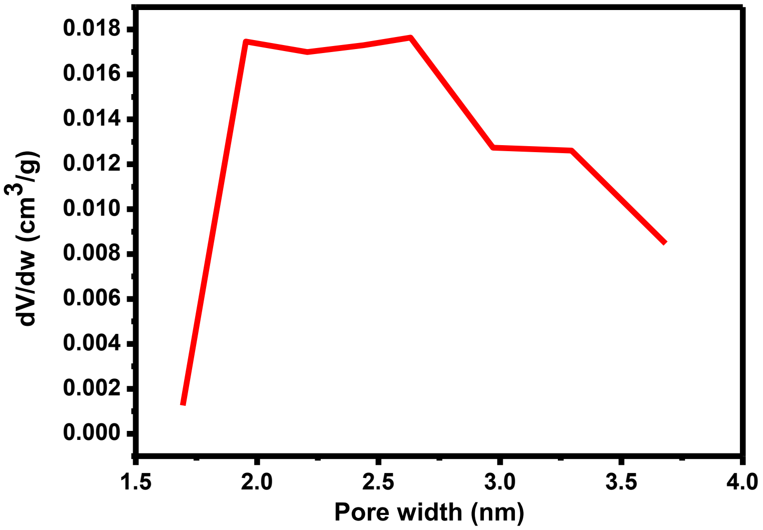

Figure S4 Horvath-Kawazoe (HK) pore size distribution curve of Fe_3O_4@CNF_{Mn}.

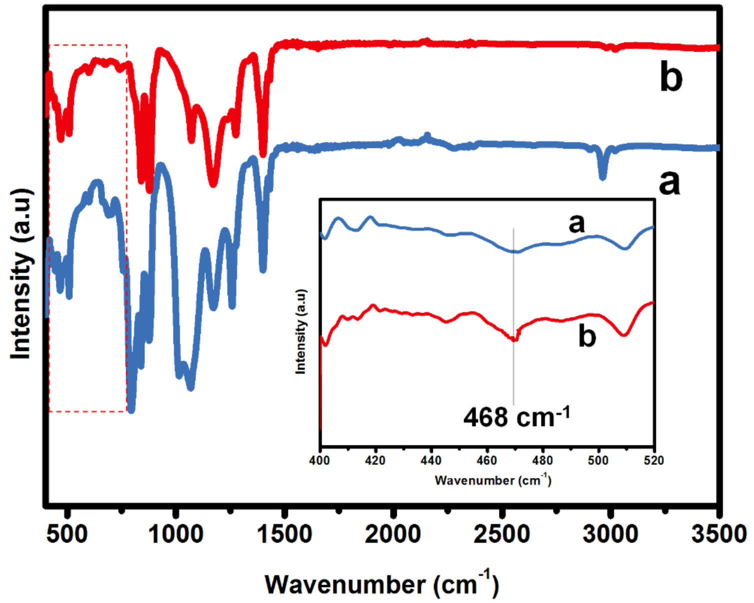

Figure S5 FTIR spectrum of Fe_3O_4@CNF_{Mn} (a) before and (b) after cycles.

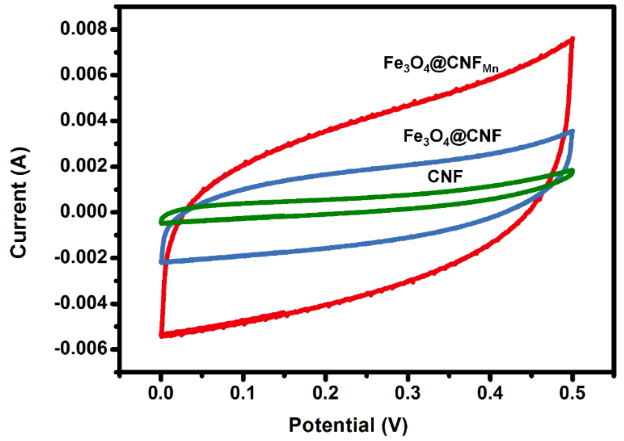

Figure S6 CV curve for pure CNF, Fe_3O_4@CNF and Fe_3O_4@CNF_{Mn} at 10 mV/s.

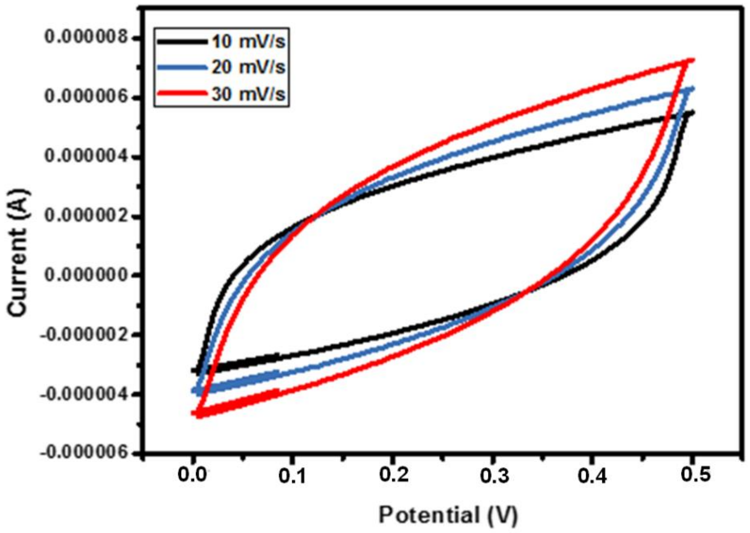

Figure S7 CV curve of Fe_3O_4@CNF.

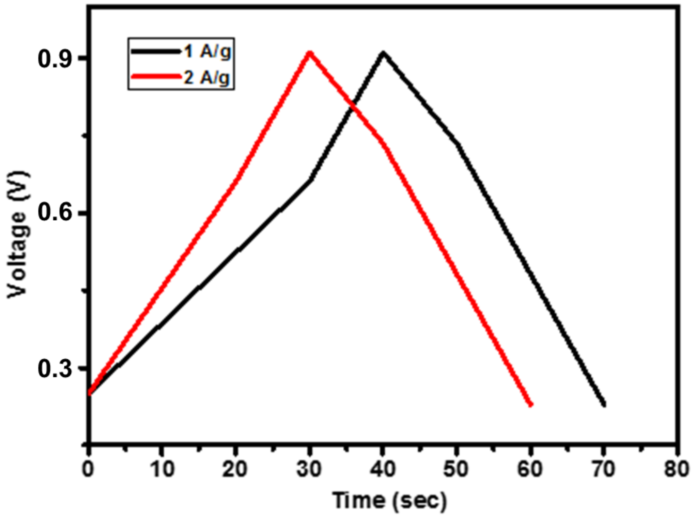

Figure S8 GCD curves of Fe_3O_4@CNF at 1-2 A/g.

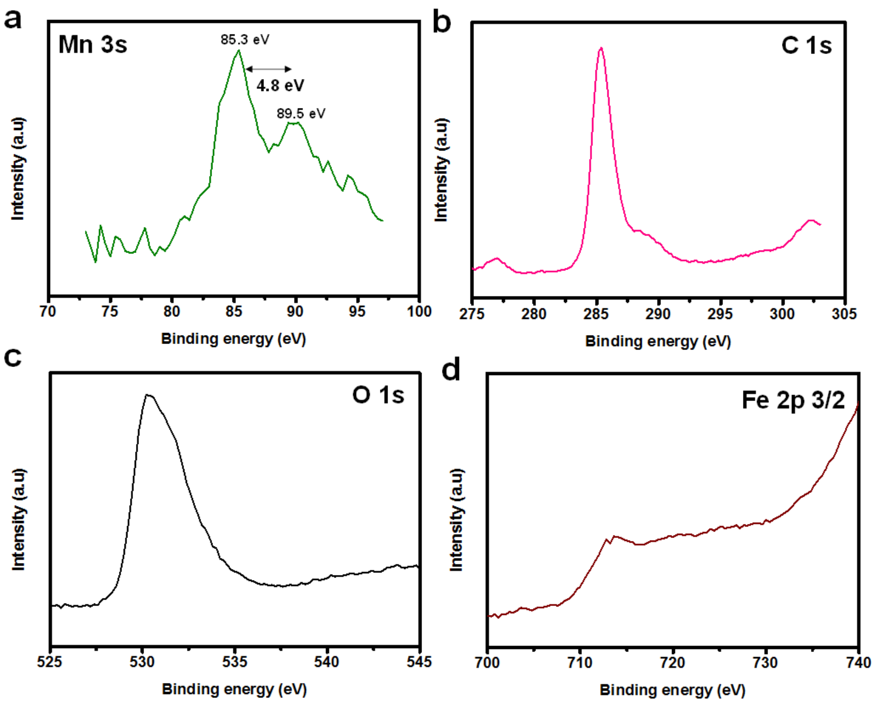

Figure S9 XPS spectra of (a) Mn 3s, (b) C 1s, (c) O 1s, and, (d) Fe 2p.

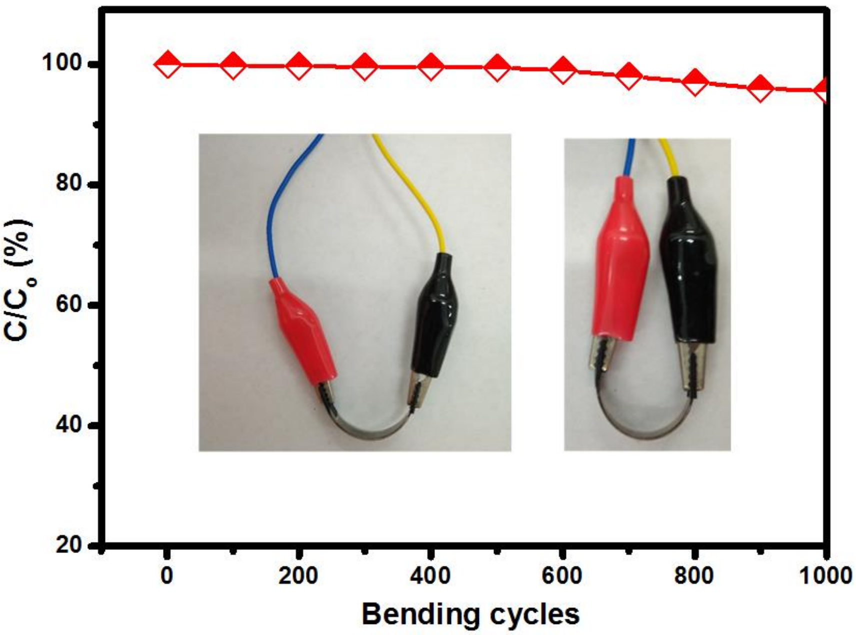

Figure S10 Dependence of specific capacitance on bending cycle number with a bending angle of 180° of Fe_3O_4@CNF_{Mn}.

Table S1. Comparison of specific capacitance between the current study and electrodes reported in the literature.

Material	Doping agent	Specific capacitance	Reference
Co_3O_4 nanostructures	Co_3O_4	202.5 F/g	1
MnO_2-coated carbon nanotubes	MnC -	193 F/g	2
Birnessite-type MnO_2	$KMnO_4$	185 F/g	3
MnO_2/graphene sheets	-	263 F/g	4
PBZ/SnO_2	SnO_2	110-118 F/g	5
Flexible Fe_3O_4@CNF_{Mn}	Fe_3O_4 and electrosprayed $KMnO_4$	306 F/g	This work

1. Wang, D., Wang, Q., Wang, T., Synthesis and hydride transfer reactions of cobalt and nickel hydride complexes to BX_3 compounds. Inorg. Chem. 50, 6482-6492, (2011).

2. Lei, Z., Shi, F., Lu, L., Incorporation of MnO_2-Coated Carbon Nanotubes between Graphene Sheets as Supercapacitor Electrode, ACS Appl. Mater. Interfaces, 4, 1058–1064, (2012).

3. Han, R., Xing, S., Ma, Z., Wu, Y., Gao Y., Effect of the $KMnO_4$ concentration on the structure and electrochemical behavior of MnO_2 J. Mater. Sci. 47, 3822–3827, (2012).

4. Li, Z., Wang, J., Liu, X., Liu, S., Ou, J., Yang, S., Evolution on Novel Graphene-based Electrode Materials for Supercapacitor, J. Mater. Chem. 21, 3397−3403, (2011).

6. Ge, J., Qu, Y., Cao, L., Wang, F., Dou, L., Yu, J., and Ding, B., Polybenzoxazine-based highly porous carbon nanofibrous membranes hybridized by tin oxide nanoclusters: durable mechanical elasticity and capacitive performance J. Mater. Chem. A 7795-7804, (2016).

Supplementary Discussion

Figure S1 depicts the FE-SEM image of precursor fibers (Fe@PAN) showing randomly oriented 3D fibrous structure which was retained even after carbonization by resultant Fe_3O_4@CNF. Figure S2 shows the cross-sectional image of Fe_3O_4@CNF which confirms the presence of randomly deposited Fe particles in the fiber matrix, whereas, Figure S3 shows Horvath-Kawazoe (HK) pore size distribution curve of Fe_3O_4@CNF_{Mn}. Figure S4 presents the FTIR spectra showing chemical characterization of the resultant Fe_3O_4@CNF_{Mn}. Moreover, electrochemical performance of the Fe_3O_4@CNF was examined by CV curves (Figure S5) and GCD curves (Figure S6) showing a low capacitive behavior.
